# Mucosal and systemic immunization against tuberculosis by ISCOMATRIX nano adjuvant co-administered with alginate coated chitosan nanoparticles

**DOI:** 10.22038/IJBMS.2023.67564.14806

**Published:** 2023

**Authors:** Adel Najafi, Kiarash Ghazvini, Mojtaba Sankian, Leila Gholami, Sirwan Zare, Arshid Yousefi Arvand, Mohsen Tafaghodi

**Affiliations:** 1 Clinical Microbiology Laboratory, Fatemieh hospital, Hamadan University of Medical Science, Hamedan, Iran; 2 Department of Microbiology, Faculty of Medicine, Mashhad University of Medical Science, Mashhad, Iran; 3 Immunology Research Center, Bu Ali Research Institute, Mashhad University of Medical Science, Mashhad, Iran; 4 Department of Molecular Medicine, Faculty of Advanced Technologies in Medicine, Iran University of Medical Sciences, Tehran, Iran; 5 Department of Laboratory Sciences, School of Allied Medical Sciences, Ahavz Jundishapur University of Medical Sciences, Ahvaz, Iran; 6 Nanotechnology Research Center, Pharmaceutical Technology Institute, Mashhad University of Medical Sciences, Mashhad, Iran

**Keywords:** Adjuvant, Chitosan, Immune response, ISCOMATRIX, Nanoparticle

## Abstract

**Objective(s):**

BCG vaccine has no longer been appreciated to immunize against tuberculosis, worldwide, so novel appropriate adjuvants have been dedicated to improve immune responses. This study aimed to evaluate the immunomodulatory effects of ISCOMATRIX as an adjuvant to stimulate potent humoral and cellular immune responses of the PPE17 loaded alginate coated nanoparticles through subcutaneous and intranasal vaccination.

**Materials and Methods:**

Size, polydispersity index, and morphology of the resulting colloidal particles were explored by dynamic light scattering (DLS). The cellular and/or humoral immune stimulation properties of ISCOMATRIX adjuvant were measured by measuring the level of IFNγ, IL-4, IL-17, and TGFβ in spleen cell cultures and IgG1 and IgG2a in serum and sIgA in nasal lavage of immunized mice, respectively.

**Results:**

The spherical cage-like particles of ISCOMATRIX adjuvant have optimal size of 59±6 nm appropriate for an immune adjuvant vaccine. ISCOMATRIX induced robust Th1 (IFN-γ) and IL-17 cytokine response also significant IgG2a and IgG1antibodies in both subcutaneous and intranasal routes and elicited mucosal sIgA response when administered intranasally. As a booster for BCG, ISCOMATRIX induced immune responses only in subcutaneous route.

**Conclusion:**

These findings indicate that ISCOMATRIX is a promising adjuvant with the potential for increasing cellular and humoral immunity both after subcutaneous and intranasal administration.

## Introduction

Tuberculosis (TB) is a worldwide health issue, based on WHO reports TB is the 13^th^ leading cause of death and the second leading infectious killer after COVID-19 (above HIV/AIDS). 

In 2020, an estimated 10 million new cases of active tuberculosis were identified worldwide consisting of 5.6 million men, 3.3 million and 1.1 million women and children, respectively (1).TB is not limited to a specific region or country and considered as a big burden and major health hazard. Although TB is considered a curable and preventable disease, the improvements of care and prevent system has not been efficient and has not reached the WHO target (2). 

The only vaccine currently in use, Bacillus Calmette-Guerin (BCG), has an efficacy spectrum ranging from 0% to 80% and provides incomplete and variable protection against disseminated pulmonary TB (3). The World Health Organization strategy target is elimination of TB deaths up to 95% by 2035, a more effectual vaccine is required for this goal.

 In pathogeneses of TB, the major change in response of dendritic cells resulted in declining the activity of T-helper-1 (Th1), Th2, Regulatory T cells (Tregs), and Th17 cells in tuberculosis (TB) infections owing to a reduction of cytokine release from antigen-presenting cells (APCs)(4).A substantial target of TB vaccination has been to create intense Th1 responses (5).

According to different studies, the use of the TB antigen alone does not have enough capability to produce enough effective protective adaptive immune response. So, adjuvant platforms and subunit vaccines may be applied to generate the adequate type of immune response essential for effective protection against a specific pathogen. Adjuvants provide T-cell co-stimulatory signals and stimulate and activate the immune response to the vaccine (5).

ISCOMATRIX, a particulate antigen delivery system with average size of 40–100 nm, represented an appropriate potential in induction of cellular and humoral immunity. (6). These cage-like structures constitute of immune stimulating complex (ISCOM), a set of adjuvants including Quil A saponin (saponin adjuvant from *Quillajasaponaria* bark, cholesterol, and phospholipids (5, 7). 

Some immunological pathways to which ISCOMATRIX adjuvant have been attributed include recruiting NK cells, lymphocytes, DC, and granulocytes to the draining lymph node following administration, and generate a Th1/Th2 (8) response Ag delivery in draining lymph node (9), implantation of DCs maturation (10), prolonged presentation of antigen in DCs due to depot effect (11), and cross presentation in antigen presenting cells (APCs) (12). ISCOMs Induction of CD8^+^T-cell response was also introduced to destroy TB bacilli through Cytotoxic T lymphocytes (CTLs) that require both immune modulatory and antigen delivery properties of ISCOMATRIX adjuvant (10, 13).

In previous studies ISCOMATRIX adjuvanted vaccines were shown to be safe and immunogenic, generating both antibody and CD4+ and CD8+ T-cell responses (10, 8). Therefore, ISCOMATRIX adjuvant is appropriate for use in prime–boost strategies as prophylactic and therapeutic vaccines (14). With regard to the increasing trend in TB incidence and mortality, investigating the functionalities of an ISCOMATRIX adjuvant co-delivered with alginate-coatedPPE17 protein-loaded chitosan nanoparticles NP(P) would be satisfactory for TB stop strategies.

## Materials and Methods

***Materials***

Cholesterol, Phosphatidylcholine and *Quillajasaponaria* bark (Quil A) were purchased from sigma chemicals company (St. Louis, USA).Chitosan (deacetylation degree of 98%) viscosity of 8 cp was received as a gift from PrimexBioChemicals AS (Avaldsnes, Norway). Low molecular weight sodium alginate and TPP (tripolyphosphate) were provided by Sigma Chemicals (St. Louis, USA). Phytohemagglutinin (PHA) and Fetal bovine serum (FBS) were from Gibco (Thermo fisher scientific, Germany). 1 M Hepes buffer (0.85% NaCl), RPMI 1640 without L-glutamine and Pen-Strep (penicillin 10,000 U /ml; 10,000 streptomycin/ml)(Sigma chemicals,St. Louis, USA). The mouse cytokine ELISA quantification Kits were from eBioscince (Thermo fisher scientific, USA). The HRP-conjugated goat anti-mouse secondary IgG1 and IgG2a and antibodies were supplied from (Thermo fisher scientific, USA). 

All other chemical solvents and reagents were chemical grade. All the solutions were prepared in deionized water.

***Vaccine preparation***

*Recombinant protein PPE17*

The PPE17 (Rv1168c) gene was PCR amplified and inserted into pET-21b(+) vector, cloned in *Escherichia* *coli *TOP10, and finally expressed in *E*. *coli *BL21(DE3). The target protein was purified by Ni–NTA column chromatography and verified by SDS-PAGE and western blot assays. The protein concentration was evaluated by BCA kit (Pars Tous Biotechnology, Iran).

*Alginate-coated chitosan nanoparticles*

The method utilized to develop alginate-coated chitosan nanoparticles was a two-step method based on an ionic gelation procedure, which was reported by our team earlier (15-17).As a short description, an aqueous solution of chitosan and TPP which contains 800 µg of recombinant PPE17 protein was prepared, and then 3 ml of TPP solution was added to 10 ml of chitosan under mild rotation. For the next step, separated chitosan nanospheres in buffer phosphate were mixed with alginate suspension at pH 7.2. At the final stage of nanoparticle synthesis, centrifuged alginate-coated chitosan nanoparticles were re-suspended and diluted in 400 µl of 0.52 mM CaCl_2 _and the resultant was exposed to bath sonication and homogenization to form uniform nanoparticles.

*ISCOMATRIX adjuvant*

The ISCOMATRIX adjuvant was prepared by hydration method in accordance with our former study with some modifications (18, 19). The synthesizing protocol was made in two distinct lipid and aqueous phases. For the preparation of lipid phase, the phosphatidyl choline (8 mg/ml) and cholesterol (4 mg/ml) solutions were dissolved in dichloromethane solvent. Then, 320 µl of phosphatidyl choline solution was mixed with 200 µl cholesterol solution and evaporated to dryness at 60 °C for 1 hr (Rotavapor, USA). After evaporation, the dried remnant was mixed with 200 mg sucrose (dissolved in 2 ml tert-buthanol and 2 ml distilled water) and evaporated again until dryness at 60 °C for 10 min. For final step, the obtained lipid film was promptly frozen by dry ice/acetone mixture and freeze-dried for 18 hr (CHRIST, Germany). In the aqueous phase, the freeze-dried lipid film was rehydrated with 4 ml PBS containing 8 mg quil A and evaporated to dryness at 60 °C for 10 min. To this end, the resulting solution was sonicated at 60 °C for 5 min (bath sonicator).

*Characteristics of ISCOMATRIX*

The particle size, zeta potential and polydispersity index (PDI) of ISCOMATRIX adjuvant was evaluated by dynamic light scattering (DLS) through a Zeta sizer instrument (Malvern, UK).All experiments were performed in triplicate and values were expressed as mean ± SD

***Animal study***

Sixty male BALB/c mice (6 to 8 weeks old age) were provided by Pasteur Institute (Tehran, Iran).The regional ethics committee of Mashhad University of Medical Sciences approved this research (NO: 931494) and all protocols and animal experiments were performed in accordance with the guidelines of Ethical Committee Acts. The cervical dislocation method was performed to dislocate the atlas cervical vertebra from the base of the skull, resulting in a rapid death.

***Immunization study***

Animals were equally divided into 10 groups for immunization investigation.

Four groups were selected as controls and other 6 groups were immunized through intranasal (IN) or subcutaneous (SC) administration. The four control groups were as follows; PBS group: 100 µg phosphate buffer solution, NP group: 10 µg blank nanoparticle, ISCO group: 12 µg Iscomatrix, and BCG group: 25x10^3^ CFU from live attenuated BCG strain. Three groups of mice were immunized intranasal (category 1) and other 3 groups injected subcutaneously (category 2). In any categories, there are three distinct groups NP (P), ISCO+NP (P), and Booster-NP(P) + ISCO. The groups NP(P) received 6 µg antigen PPE17 encapsulated by alginate coated chitosan nanoparticles, group ISCO + NP(P) and group Booster- NP(P) + ISCO simultaneously received 6 µg antigen PPE17 encapsulated by alginate coated chitosan nanoparticles along with 12 µg Iscomatrix.

Animals were immunized 3 times on days 0, 14, and 28, except for control groups PBS and BCG which received a single dose and booster groups which received BCG at day 0 followed by two booster doses of nanoparticles at days 14 and 28.

***Humoral immunity assessment***

Two weeks after the last administrations, blood samples were collected from vaccinated mice by method orbital sinus puncture. The serums were separated from blood samples and exploited for humoral assessment through IgG1 and IgG2a titration. For evaluating IgA titers in nasal lavages, the mice were euthanized by cervical dislocation and then the nasal lavages were collected after washing the nasal cavity with 1 ml of normal saline. 

The method of antibody titration was performed by end-point titration ELISA (eBioscience, USA) according to the manufacturé s instructions. To this end, specific IgG1 and IgG2a serum antibodies and nasal sIgA were measured by titration assay utilizing HRP-conjugated goat anti-mouse secondary antibodies (20). 

Accordingly, microtiter plates were coated with 2 μg/well with specific antigens in 100 µl bicarbonate buffer (pH 9.6) overnight at 4 °C. The plates were washed five times with PBS containing 0.05% Tween 20. The plates were blocked with 200 µl/well of blocking buffer (1% bovine serum albumin, BSA, and 0.5% PBS buffer with a detergent such as Tween 20) and incubated for 1 hr at 37 °C and washed 5 times with PBST. After washing, the plates were added 100 µl/well of serial dilution of serum samples and incubated at 37 °C for 2.5 hr. After washing, the plates were added 100 μl/well of 1:500 dilution of IgG1 and IgG2a secondary antibodies and then incubated for 2 hr at 37 °C. After five times washing, 100 μl/well of TMB (3, 3’, 5, 5’-tetramethylbenzidine) solution was added and incubated for 15 min at room temperature. The reaction was stopped by adding 50 μl of H_2_SO_4_ in each well and finally absorptions were read at 450 nm by a microplate reader.

***Preparation of spleen cell suspensions***

Mice are euthanized by cervical dislocation and their spleens are aseptically removed. Individual spleen cell suspensions are prepared in Petri dishes using curved needles. One needle is used to hold the spleen and the other to detach cells from the capsule by moving the needle along the length of the spleen. The cell suspension is then transferred into a 15-ml sterile conical tube to allow large fragments to settle down for 5 min. The cell suspension is decanted homogenized by 70-μm cell strainer to make cell suspension and centrifuged for 10 min at 259 × g. The resultant supernatant is discarded and cells are resuspended in 5 ml of RPMI 1640. This washing step is repeated two times, and finally, the cells (2x10^6^ cell/well) are suspended in complete RPMI 1640 medium (supplemented with 10% (v/v) fetal bovine serum (Gibco, UK), 1% penicillin/ streptomycin (Biosera, UK) and 5 µg/ml of PPE17 protein or phyto hemagglutinin (PHA) (Gibco, UK) as mitogen. 

***Cellular immunity assessment***

The cellular immune responses were evaluated through determining the level of cytokines stimulated in spleen cell cultures of vaccinated mice. After 72 hr of incubation (37 °C , 5% CO_2_) of spleen cell incubation, the level of cytokines IFN-γ, IL-4, IL-17 and TGFβ in supernatants of cell cultures were measured by sandwich ELISA method (eBioscience, USA) based on manufacturer’s guidelines. 

The steps to ELISA method after preparing the samples were as follows: coating the specific antibodies specific for the desired antigens within the microval, adding sample liquid tissue and the standard liquid to attach the antigen to the coated antibodies, adding biotin conjugate, washing out the extra protein not bonded with antigens by wash buffer, adding conjugated and labeled with the HRT enzyme specific antibody to microval and adding substrate solution and colored material. After this stage for the presence of the enzyme (HRT), substrate was converted to product; the amount and intensity of the color in this conversion was proportional to the amount of antibody in the standard tissue and the samples. Then, the reaction was ended by stop solution which was an acid and the amount of its light absorption was 450 nm and then, standard dilution rate and sample concentration were determined by using the prepared standard curve for IL-4, IL-17, and IFNγ cytokines. The amount of IL-4, IL-17, and IFNγ then was measured in picograms (pg) and the result was determined by standard curve

***Statistical analysis***

Data were expressed as mean ± standard error of the mean (SEM). All data analysis was performed by Two-way ANOVA Tukey’s multiple comparison tests. *P*-value<0.05 was considered significant, in group comparison. Significance was presented as *(*P*<0.05), ** (*P*<0.01), ***(*P*<0.001), and ****(*P*<0.0001).

## Results

***PPE17 loaded alginate coated chitosan nanoparticles***

Here in our study, PPE17 loaded alginate coated chitosan nanoparticles were formulated according to the same procedures investigated earlier by Najafi *et al*. As a result, the NPs were 427 nm in size with a -37 mv surface charge due to coating with alginate.The encapsulation efficiency of NPs was 11% and the release pattern showed a steady release of antigens from NPs that lasted to 144 hr* in vitro* (15).

***Characteristics of ISCOMATRIX adjuvant***

As mentioned before, ISCOMATRIX adjuvant was prepared by the hydration method. The structure and characteristics of synthetic adjuvant ISCOMATRIX were consistent with the features appropriate for immune-adjuvant properties. The cage-like structure of ISCOMATRIX has an optimal size of 59±6 nm (n=3) with zeta potential of -9.1 ± 0.4 (n=3). Likewise, the polydispersity index (PDI) concomitant with other outlined results was 0.33 ± 0.06 (n=3).

***Evaluation of immune response***

*Humoral immunity*

As shown in Figure 2, ISCOMATRIX has increased the specific anti PPE17 serum antibodies IgG1 and IgG-2a and mucosal sIgA after both SC and IN vaccination. Indeed, the level of IgG2a stimulation for group NP (P) + ISCOMATRIX is much higher than NP (P) group. On the other, the NP (P) + ISCOMATRIX group has stimulated IgG1 antibody response equal to the NP (P) group. Mucosal antibody sIgA as an index of nasal protection was stimulated in both SC and IN routes, but the IN route has triggered sIgA more impressively.

***Cellular immunity***

For evaluating cellular immune responses, the levels of cytokines IFN-γ, IL-17, TGF-β, and IL-4 were detected in the supernatant of cultured mice splenocytes after inducing with antigen PPE17. The increase in the IFN-γ response, as shown in Figure 3A, was significantly triggered by co-administration of ISCOMATRIX with alginate-coated chitosan nanoparticles through both SC and IN vaccination and it was stronger after nasal administration (**** *P*<0.0001). Like IFN-γ, the same pattern was observed for IL-17 Figure 3b and IL-4 Figure 3c responses. But the nasal route showed poor responses of IL-17 and IL-4 stimulations compared with SC route.

**Figure 1:**
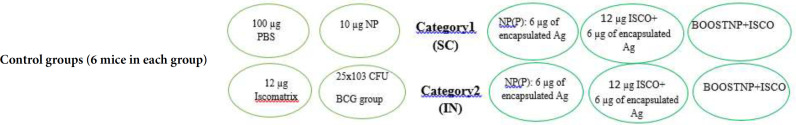
Schematic design of animal study. Sixty BALB/c mice were divided in 10 groups

4 groups (6 mice in each group) were selected as controls and other 6 groups were immunized through intranasal (IN) or subcutaneous (SC) administration. The four control groups were as follows; PBS group: 100 μg phosphate buffer saline, NP group: 10 μg blank nanoparticles, ISCO group: 12 μg Iscomatrix, and BCG group: 25x103 CFU from live attenuated BCG strain.

Three groups of mice were immunized intranasal (category 1) and other 3 groups injected subcutaneously (category 2).

In any categories, there are three distinct groups NP (P), ISCO+NP (P), and Booster-NP(P) + ISCO. The groups NP(P) received 6 μg antigen PPE17 encapsulated with alginate

coated chitosan nanoparticles, group ISCO + NP(P) and group Booster- NP(P) + ISCO simultaneously received 6 μg antigen PPE17 encapsulated by alginate coated chitosan

nanoparticles along with 12 μg Iscomatrix.

**Figure 2:**
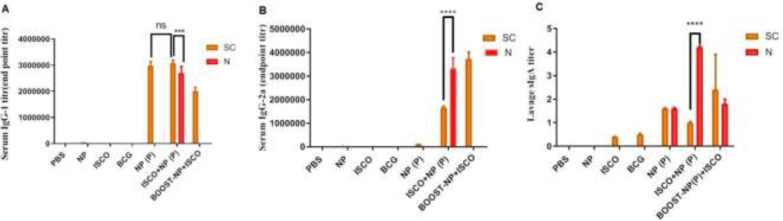
Serum titration of IgG-1(A), IgG-2a (B), and sIgA (C) antibody was evaluated after two weeks of intranasal and subcutaneus immunization

BALB/c mice were treated with different alginate coated and PPE17 loaded chitosan nanoparticles using the ELISA method. Results are expressed as the mean value±SEM (n=5). * *P*<0.05, ** P<0.01, *** P<0.001, and **** *P*<0.0001

**Figure 3:**
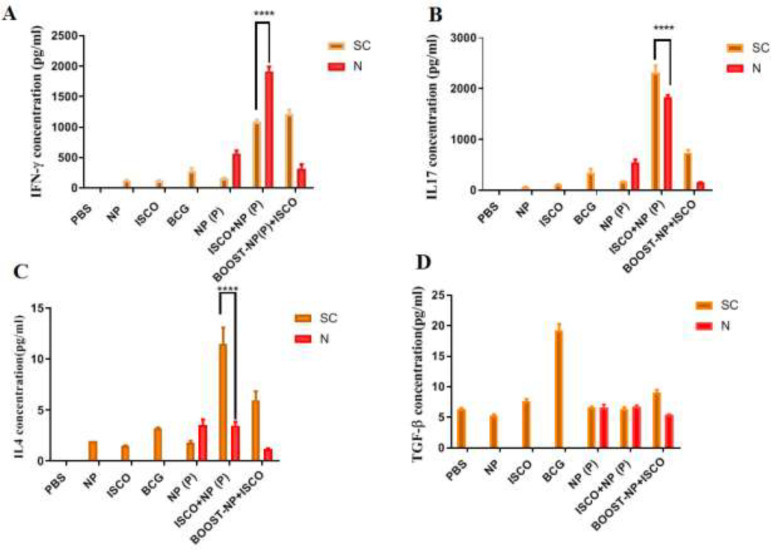
The level of IFN-γ (a), IL-17 (b), IL-4 (c), and TGF-β (d) production after two weeks (the last immunization) of intranasal and subcutaneus immunization

BALB/c mice were received different alginate coated and PPE17 loaded chitosan nanoparticles and titer of TGF-β (d) was determined from the cultured splenocytes using the ELISA method. Results are expressed as the mean value±SEM (n=5). * *P*<0.05, ** *P*<0.01, *** *P*<0.001, and **** *P*<0.0001.

## Discussion

ISCOMATRIX adjuvanted vaccines have been investigated for a number of infectious diseases including tuberculosis (21). Moreover, intranasal administration of ISCOMATRIX associated TB vaccine has been shown to generate robust IFN-γ stimulation as a cellular immunity profile (22). ISCOMATRIX adjuvanted vaccines are appointed to generate strong and long-lasting CD4+ and CD8+ T-cell responses that resulted in the induction of strong immune responses (23). Here, Th1 immune stimulation by ISCOMATRIX has been demonstrated by linking innate and adaptive immune responses through DC maturation and Ag presentation in vaccine site-draining lymph nodes (23). Furthermore, a marked increase of IFN-γ from NK cells followed by incorporating T and B cells has been suggested to play a critical role in immune activation by ISCOMATRIX. Cross-presentation, the process by which Ag can be represented to MHC class I molecules for activating CD8+ T-cell, are being accounted as a highly specialized function of DCs dealing with ISCOMATRIX adjuvant immunization (12).

Overall, ISCOMATRIX has been indicated as a potent immune modifier by which CD8+ T-cell immune responses are being modulated (23).

Hereby, we investigated how ISCOMATRIX adjuvant could enhance immune responses through both intranasal and subcutaneous routes of immunization as prime-boost strategies.We found that ISCOMATRIX adjuvant could induce potent cellular-related cytokines IFN-γ and IL-17 through IN and SC routes.

As shown in Figure 1, addition of ISCOMATRIX adjuvant to PPE17 protein appeared to enhance IFN-γ and IL-17 responses through IN and SC routes. Moreover, our results revealed that immunization of animal models with NP+ISCO as a booster vaccine can enhance cellular immune responses through SC route rather than IN route. however, IL-4 as Th2 type immune response factor was induced through SC administration rather than IN route. This discrepancy between the levels of IFN-γ and IL-4 stimulation amongst SC and IN groups was implied from the fact that the profiles of cytokines are different in each type of antigen delivery into immune systems.

In this regard, the induction of humoral immunity can also have confirmed the immune adjuvant effects of ISCOMATRIX. As shown in Figure 2, high levels of IgG2a responses were reported for nasal administration of ISCOMATRIX followed by SC routes as a booster that has acceptable correlations with Hu et al. reports (24).The increased level for IgG-2a was seen only for subcutaneous use of booster vaccine, which was also reported for IFN-γ and IL-17. For both cellular immunity cytokines, the booster vaccine showed better results in SC administration.

The serum IgG1 responses for SC-delivered ISCOMATRIX were significantly higher than the nasal-delivered booster vaccine but in the case of particulated PPE17 prime antigen +ISCOMATRIX the differences between nasal and SC response were significant *P*≤0.001.

It seems that booster vaccine NP+ISCO can provide effective Th2 and Th1 type immune responses only in the SC mode.

Mucosal immunity is characterized by the production of secretory IgA(sIgA), referring as the advantages of intranasal vaccine delivery (25).We found that ISCOMATRIX adjuvant can induce sIgA responses as a prime vaccine and also as a booster in both IN and SC use of vaccines but the higher stimulation was evaluated with intranasal administration which seems logical.

Indeed, our results confirm the previous reports that the immunization route and nature of antigen could modulate the type of immune responses elicited by an adjuvant (26). Likewise, consistent with several reports based on inducing Th1 immune responses through subcutaneous and also Th2 bias immune responses through intranasal administration (8, 27), our data indicated that the nature of antigen and strategies of immunization as prime-boost vaccination are two impressive issues that are not to be neglected. To our knowledge, this is the first study that reports that ISCOMATRIX adjuvant is specifically able to modulate immune responses through influencing and selecting Th1 and/or Th2 immune responses depending on which strategies of prime-boost vaccination was utilized. The results obtained from this work could support our hypothesis that addition of ISCOMATRIX adjuvant to PPE17 protein could enhance IFN-γ and IL-17 responses through IN and SC routes, therefore it confirmed the adjuvant and Ag delivery properties of ISCOMATRIX adjuvant.

## Conclusion

A well-known mechanism of improving the immunogenicity of vaccines involves the incorporating of adjuvants. Iscomatrix adjuvants are considered impressive tools to improve nanovaccine immune response. In this study the effectiveness of iscomatrix adjuvants on improving and modulating Th1 and IL-17 immume responses in mice were investigated. This study indicated that iscomatrix could induce stronger IFN-γ and Th-17 stimulation in animal models. 

## Authors’ Contributions

A N carried out the study and drafted the manuscript. A N and S Z provided data acquisition and analysis and assisted in statistical analysis. A YA participated in data collection. L G interpreted the data for the work and revised the drafted manuscript for important intellectual content. K G and M S participated in study design and coordination. M T conceived and designed the work and revised the manuscript. All authors read and approved the final manuscript.

## Funding

The authors sincerely acknowledge the financial support of Mashhad University of Medical Sciences, Iran to perform this study.

## Statement of Human and Animal Rights

The study protocol was approved by the ethical committee of Mashhad University of Medical Sciences, Iran.

## Competing Interests

The authors state that they have no competing interests.
